# Postbiotics: Functional Food Materials and Therapeutic Agents for Cancer, Diabetes, and Inflammatory Diseases

**DOI:** 10.3390/foods13010089

**Published:** 2023-12-26

**Authors:** Sangiliyandi Gurunathan, Pratheep Thangaraj, Jin-Hoi Kim

**Affiliations:** 1 Department of Biotechnology, Rathinam College of Arts and Science, Eachanari, Coimbatore 641021, Tamil Nadu, India; pratheep.bio@rathinam.in; 2 Department of Stem Cell and Regenerative Biotechnology, Konkuk University, Seoul 05029, Republic of Korea

**Keywords:** postbiotics, prebiotics, probiotics, metabolites, lactic acid bacteria, fermentation

## Abstract

Postbiotics are (i) “soluble factors secreted by live bacteria, or released after bacterial lysis, such as enzymes, peptides, teichoic acids, peptidoglycan-derived muropeptides, polysaccharides, cell-surface proteins and organic acids”; (ii) “non-viable metabolites produced by microorganisms that exert biological effects on the hosts”; and (iii) “compounds produced by microorganisms, released from food components or microbial constituents, including non-viable cells that, when administered in adequate amounts, promote health and wellbeing”. A probiotic- and prebiotic-rich diet ensures an adequate supply of these vital nutrients. During the anaerobic fermentation of organic nutrients, such as prebiotics, postbiotics act as a benevolent bioactive molecule matrix. Postbiotics can be used as functional components in the food industry by offering a number of advantages, such as being added to foods that are harmful to probiotic survival. Postbiotic supplements have grown in popularity in the food, cosmetic, and healthcare industries because of their numerous health advantages. Their classification depends on various factors, including the type of microorganism, structural composition, and physiological functions. This review offers a succinct introduction to postbiotics while discussing their salient features and classification, production, purification, characterization, biological functions, and applications in the food industry. Furthermore, their therapeutic mechanisms as antibacterial, antiviral, antioxidant, anticancer, anti-diabetic, and anti-inflammatory agents are elucidated.

## 1. Introduction

Food stands as a fundamental human requirement crucial for sustaining all aspects of life. Nutrients such as lipids, carbohydrates, proteins, fibers, phytochemicals, antioxidants, vitamins, probiotics, and prebiotics provide different health benefits [1,2,3,4]. Whole grains and dietary fiber serve as rich sources of vitamins, minerals, and slowly digestible energy. Intense innovation in the functional food industry has led to the production of a vast spectrum of health-promoting bioactive components, including prebiotics, phytochemicals, natural antioxidants, and bioactive peptides [5]. Emerging food technologies are actively used to process food products, resulting in increased probiotic functionality, prebiotic stability, and higher concentrations of bioactive compounds [6].

Probiotics, as defined by the Food and Agriculture Organization of the United Nations–World Health Organization (FAO-WHO), are “live microorganisms that, when administered in adequate amounts, confer a health benefit on the host” [7]. The majority of probiotic products contain a defined and constrained list of microbial taxa, primarily lactic acid bacteria (LAB) like *Lactobacillus* spp.and *Bifidobacterium* spp., recognized as generally safe (GRAS) [7]. While yogurt is a common carrier of probiotics, other fermented (cheese and yogurt) and non-fermented (cereal, chocolate bars, fruit juices, and smoothies) meals may also incorporate probiotics. Probiotics impact the gut microbiota by suppressing infections, inhibiting their adherence to tissues, and impeding their establishment in the gut [8,9]. Furthermore, probiotics contribute to immune system development, the production of essential nutrients, and the reinforcement of intestinal barrier integrity by activating genes linked to tight junction signaling [9].

According to a consensus statement by the International Scientific Association of Probiotics and Prebiotics, “Prebiotics is a substrate that is specifically utilized by host microorganisms to provide a health advantage”. Prebiotics, found in dietary fibers, phenolics, phytochemicals, conjugated linoleic acids, polyunsaturated fatty acids, human milk oligosaccharides (HMOs), and various oligosaccharides, can alter the composition of the microbiota by promoting the growth of specific species, thereby benefiting the host’s health [10,11]. A synbiotic, commonly defined as a synergistic mixture of probiotics and prebiotics, beneficially affects the host by improving the survival and colonization of live beneficial microorganisms in the gastrointestinal tract [12,13].

Postbiotics are “soluble factors secreted by live bacteria, or released after bacterial lysis, such as enzymes, peptides, teichoic acids, peptidoglycan-derived muropeptides, polysaccharides, cell-surface proteins and organic acids”; (ii) “non-viable metabolites produced by microorganisms that exert biological effects on the hosts”; and (iii) “compounds produced by microorganisms, released from food components or microbial constituents, including non-viable cells that, when administered in adequate amounts, promote health and wellbeing”. These molecules mediate beneficial biological activities directly or indirectly when administered to consumers [14,15,16,17,18]. Described as “preparations of inanimate microorganisms and/or their components that confer a health benefit to the host”, postbiotics are produced by bacterial and fungal species like *Lactobacillus*, *Bifidobacterium*, *Streptococcus*, *Eubacterium*, *Faecalibacterium*, and *Saccharomyces*, which are naturally found in fermented foods such as yogurt, sauerkraut, pickled vegetables, and kombucha [19,20,21]. Several commercial postbiotics, primarily used to treat gastrointestinal or immune-related disorders, are available as supplements or in food matrices [22]. Comprising bacterial lysates with cell surface proteins, bacterial enzymes, peptides, metabolites, and lower organic acids like lactic acid, postbiotics derive their efficacy from microbial metabolites, proteins, lipids, carbohydrates, vitamins, organic acids, cell wall components, and other complex compounds produced in the fermented matrix [20,23]. Food processing techniques, including heat, sonication, irradiation, and high pressure, can impact postbiotic composition, with potential effects on the microorganisms involved in fermentation [24,25,26,27,28,29,30,31,32,33,34]. Various analytical techniques such as matrix-assisted laser desorption/ionization time-of-flight (MALDI-TOF) mass spectrometry and HPLC are suggested for postbiotic identification [35,36,37]. Studies have assessed the bioactivity and health effects of postbiotics, including intracellular metabolites and cell wall components, both in vitro and in vivo [38]. Dairy-based items, like kefir, kombucha, yogurt, and pickled vegetables, naturally contain postbiotics [39].

Producer strains, primarily *Lactobacillus* and *Bifidobacterium* varieties, and other species like *Streptococcus*, *Akkermansia muciniphila*, *Eubacterium hallii*, *Faecalibacterium*, and *Saccharomyces boulardii*, can be used to extract postbiotics in situ. Milk products containing exopolysaccharides (EPSs) made by lactic acid bacteria (LAB), particularly *Lacticaseibacillus rhamnosus*, enhance the physicochemical and sensory properties of food-based products [20,40]. *Streptococcus* and *Faecalibacterium* species are also potential sources of postbiotics [14,23]. Postbiotic supplements, primarily produced by *Lactobacillus* and *Bifidobacterium* spp., have been shown to lower blood pressure, indicating their potential in treating hypertension [38].

As highlighted by Klemashevich [41], the intestinal microbiota influences various gastrointestinal tract processes, including inflammation, pathogen defense, and immune system development. A study on *Lactobacillus helveticus* MB2-1 demonstrated the potent capacity of the crude culture extract and pure EPS to scavenge three different types of free radicals and chelate ferrous ions [42]. This review offers a concise history and explanation of postbiotics, covering their classification, manufacturing, purification, and characterization, along with insights into their biological activities and potential applications in the food industry.Furthermore, therapeutic mechanisms of postbiotics as antibacterial, antiviral, antioxidant, anticancer, anti-diabetic, and anti-inflammatory agents are discussed.

## 2. History and Concept of Postbiotics

More than 2000 years ago, Hippocrates asserted that “all disease begins in the gut”. Today, there is no doubt that the intestinal microbiome plays a critical role in the pathogenesis of various systemic autoimmune and inflammatory disorders [43]. The emergence of studies on intestinal flora was initiated by the Russian microbiologist Elie Metchnikoff, who began working with Louis Pasteur at the Pasteur Institute in Paris in the early 1900s. Louis Pasteur (1822–1895) made significant advancements in pasteurization, microbial fermentation, and vaccination, and Louis Pasteur was the inventor of these procedures. During his lifetime, he developed treatments for several of the deadliest diseases worldwide, including rabies, anthrax, tuberculosis, cholera, and smallpox. It has been suggested that discovering treatments for cancer, heart disease, diabetes, and Alzheimer’s disease would be the modern-day equivalent of his accomplishments. In addition to Pasteur’s remarkable achievements, he contributed to the germ theory of disease by popularizing the idea that germs cause disease. Consequently, antibiotic medications were developed, leading to a century of bacterial fear. Over the past several decades, the overuse of antibiotics has damaged the immune system, destroyed the microbiome, and given rise to infectious diseases with lethal “superbug” antibiotic resistance.

**Figure 1:**
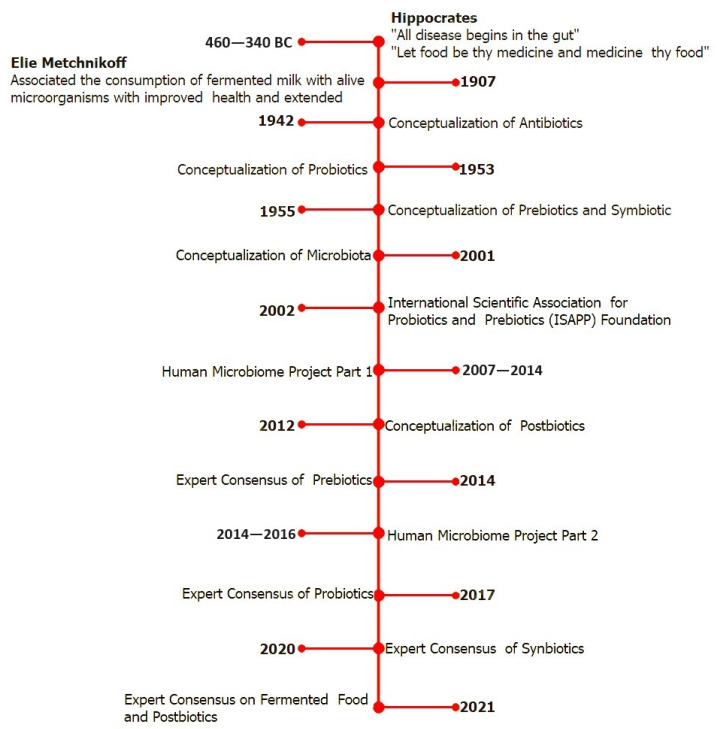
Schematic diagram illustrating the major historical milestones in the development of prebiotics, probiotics, synbiotics, and postbiotics.

In the early 1900s, Metchnikoff developed a keen interest in understanding how and why people age while working at the Pasteur Institute. During the early 1900s, Bulgaria boasted an extraordinarily long average lifetime of 87 years, with four out of every 1000 people over 100 years old, according to figures collected during Metchnikoff’s study of 36 nations. Metchnikoff hypothesized that “bad” bacteria in the intestinal tract, producing toxins, were responsible for the aging process. He attributed this to what he termed “intestinal auto-intoxication”, leading to age-related bodily deterioration and breakdown [44]. Metchnikoff ultimately developed a profound intuitive understanding that earned him the title of the “Founding Father of Probiotics”. According to Metchnikoff, the daily consumption of fermented milk products, such as yogurt and kefir, accounted for the long and healthy lives of Bulgarians. He recognized that lactic acid produced by bacteria in milk during fermentation created an acidic environment in the gastrointestinal tract (GI tract). Metchnikoff postulated that this environment prevented the formation of “bad” microorganisms, resulting in less “intestinal auto-intoxication”, improving health, and extending lifespan (Figure 1).

The current state of microbiome science has significantly benefited from the development of postbiotics. The collection of microorganisms, including bacteria, fungi, and viruses, residing inside and on our bodies is known as the “human microbiome”. The first successful sequencing of the human genome was undertaken by the Human Genome Project, with a budget of USD3 billion over 13 years (1990–2003). This paved the way for cures for many chronic degenerative diseases. However, the Human Genome Project also led to technology allowing researchers to sequence genomes rapidly and affordably. The Human Microbiome Project, resulting in the publication of over 350 articles, is considered the “birth” of the modern age of microbiome science.

Our ancestors recognized the vital role fermented foods and bacteria play in health, especially gut health [45]. The health of a host is significantly influenced by the gastrointestinal microbiota (GM) due to the protective roles of microorganisms [46]. Microbial dysbiosis, or an imbalance in microbial populations, has been linked to various human disorders, such as obesity, diabetes, colon cancer, inflammatory bowel diseases, neurological issues, psychiatric difficulties, and allergies [47,48,49,50,51]. The GM seems to contribute to the onset and course of disease in these circumstances [52,53,54]. Probiotics and prebiotics play a crucial role in repairing intestinal microbial habitats and thus benefiting health [55,56]. Numerous studies have shown that strategies for altering GM composition or activity, such as supplementation with these substances, are highly effective. Most studies on GM modulators have focused on probiotics and prebiotics. Living bacteria, known as probiotics, support host health by improving intestinal barrier function, infection defense, and immune response modulation [9,11]. Among bacteria, species from the genera *Lactobacillus*, *Bifidobacterium*, *Streptococcus*, and *Lactococcus*, as well as yeasts from the genus *Saccharomyces*, are most frequently used [57,58,59]. Postbiotics are live microorganisms that, when administered to a host in sufficient quantities, impart health benefits [60]. Prebiotics are substrates specifically utilized by host microbes and confer health benefits to the host [11]. A symbiotic is defined as a combination of live microorganisms and substrates that are utilized by host microorganisms only when advantageous to host health [61]. Until recently, the term mostly referred to indigestible fibers, but its modern meaning now encompasses bioactive substances from various sources, such as polyunsaturated fatty acids and polyphenols.

Technological advances have enabled the examination, categorization, and characterization of different probiotic-related concepts. The International Scientific Association for Probiotics and Prebiotics (ISAPP), among others, has established an expert consensus and aids in the development of novel concepts. In 2019, ISAPP assembled a panel of specialists to evaluate the definition and use of postbiotics [62,63]. These specialists, with expertise in nutrition, microbial physiology, gastroenterology, pediatrics, food science, and microbiology, defined postbiotics as “the preparation of inanimate microorganisms and/or their components that confer a health benefit on the host”. Effective postbiotics must inactivate microbial cells or cell components, with or without metabolites, to contribute to claimed health benefits [62]. Postbiotics, referred to as “biogenics” by Mitsuoka in 1998, have been accessible in Japan for over a century. It is essential to highlight the most recent changes in the ISAPP consensus statement regarding the definition and scope of postbiotics, in addition to new biotic terminology [18]. The ISAPP defined postbiotics as (i) “soluble factors secreted by live bacteria, or released after bacterial lysis, such as enzymes, peptides, teichoic acids, peptidoglycan-derived muropeptides, polysaccharides, cell-surface proteins and organic acids”; (ii) “non-viable metabolites produced by microorganisms that exert biological effects on the hosts”; and (iii) “compounds produced by microorganisms, released from food components or microbial constituents, including non-viable cells that, when administered in adequate amounts, promote health and wellbeing”.

The concept of postbiotics is relatively recent compared with that of prebiotics and probiotics. Postbiotic supplements, despite not being widely available, are superior to probiotics. This is due to their purity, ease of preparation, long shelf life, large-scale production feasibility, precise actions, and the ability to trigger more focused responses through specific ligand–receptor interactions [64].

Experimental studies suggest that microbial components, distinct from their live counterparts (probiotics), are desirable subjects for investigation. Postbiotics, a group that has recently garnered much attention, exhibit therapeutic effects not restricted to the intestine alone. These effects should be assessed or proven in humans, animals, and other target organs. The panel concluded that postbiotics are intentionally inactivated bacteria containing metabolites or cellular components with positive health effects. Additionally, the target population, including people, pets, and cattle, must derive local and systemic health advantages. The injection of postbiotics onto the host surface appears to be safe and favorable. Postbiotics were defined to exclude pure microbial metabolites and vaccines; however, probiotics are not always a source of antibiotics.

Postbiotics have demonstrated positive immunomodulatory, antibacterial, and anticancer effects, including lowering blood pressure, cholesterol levels, proliferative qualities, inflammation, oxidative stress, and body weight. Healthy individuals tolerate postbiotics well. However, some individuals refrain from consuming probiotic-rich meals to elevate their postbiotic levels, such as those who have recently undergone surgery, those with structural heart abnormalities, those with digestive tract illnesses, pregnant women, and young children. Due to their weakened immune systems, these groups may be more susceptible to negative reactions. Several foods, including high-fiber meals such as oats, flaxseed, and garlic, as well as buttermilk, cottage cheese, fermented pickles, and yogurt, may aid in the gut’s postbiotics. Numerous studies have reported that postbiotic performance may be influenced by both internal and external factors. Interactions between active postbiotic metabolites and internal elements, such as existing microbiota, enzymes, and different dietary components, can inhibit the ability of metabolites to function [65]. Proteolytic enzymes, including pepsin, trypsin, and chymotrypsin, have been associated with postbiotic dysfunction [66,67,68]. Temperature is another external factor influencing the antibacterial activity of postbiotics. According to Mirnejad [69], heat treatment for 30 min at 121 °C reduces the antibacterial efficacy of postbiotics. Therefore, maintaining consistent temperature and pH is essential for the formation of postbiotics.

## 3. Characteristic Features of Postbiotics

According to ISAPP, a postbiotic is defined as (i) “soluble factors secreted by live bacteria, or released after bacterial lysis, such as enzymes, peptides, teichoic acids, peptidoglycan-derived muropeptides, polysaccharides, cell-surface proteins and organic acids”; (ii) “non-viable metabolites produced by microorganisms that exert biological effects on the hosts”; and (iii) “compounds produced by microorganisms, released from food components or microbial constituents, including non-viable cells that, when administered in adequate amounts, promote health and wellbeing” [11]. Postbiotics are compounds formed after microbes have been rendered inert, dead, or inactivated. In contrast, postbiotics are probiotic-derived products made from food-grade bacteria that, when taken in sufficient doses, have positive effects on health [70]. They consist of cellular components and secreted substances, metabolic waste released by living microorganisms, or are gathered and extracted during cell lysis [16]. The bacteria that constitute a postbiotic can be whole, inert, or broken down into their structural components, such as cell walls. Many postbiotic preparations contain chemicals produced by microbes, such as metabolites, proteins, and peptides. These substances may provide a postbiotic’s overall health benefits but are not required. A postbiotic must originate from a specific microorganism or a combination of microorganisms whose genomic sequences are known and can be created using a well-defined technological process of biomass generation and inactivation that is repeatable [71]. Although postbiotics of fungal origin are also being studied, the majority are formed from bacteria, most frequently *Lactobacilli* and *Bifidobacterium* members. Currently, some commercial postbiotics are available as supplements or included in food matrices, most of which are used to treat gastrointestinal or immune system-related disorders [18,20]. Lipoteichoic acid extracted from *L. plantarum* significantly attenuates *Shigella flexneri* PGN (flexPGN)-induced pro-inflammatory signals in human monocytic THP-1 cells [72]. Oral administration of *Faecalibacterium prausnitzii* supernatant decreased the severity of 2,4,6-trinitrobenzene sulfonic acid (TNBS)-induced colitis in mice and corrected the dysbiosis associated with TNBS colitis, highlighting its potent anti-inflammatory effects [33]. *Saccharomyces cerevisiae* is a well-known probiotic-producing yeast used for the bioproduction of metabolites and serves as an anti-inflammatory component [73]. Human polymorphonuclear (PMN) cells treated with cell wall fragments and metabolites obtained from *Bacillus coagulans* inhibited oxidative stress-induced reactive oxygen species (ROS) formation [73].

**Figure 2:**
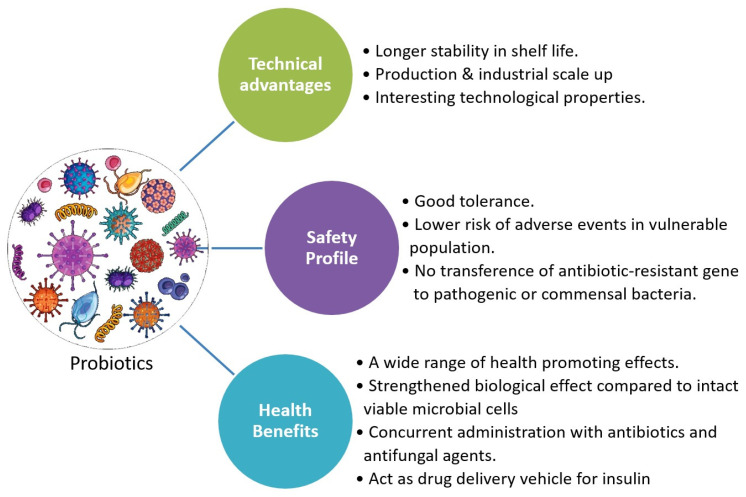
Scheme illustrating the prominent technical advantages, safety profile, and health benefits of utilizing postbiotics.

Postbiotics have several noteworthy characteristics, such as (i) being relatively safe, (ii) being well-tolerated and related to a decreased risk of adverse effects in vulnerable persons [14,25,74,75], and (iii) being quite safe. According to Zókiewicz et al. [76], they do not carry the risk of spreading antibiotic-resistant genes to commensal or pathogenic bacteria, and (iv) their efficacy is independent of cell viability, resulting in increased stability and shelf life. (v) They demonstrate straightforward industrial (large-scale) production [64]; (vi) they exhibit intriguing technological characteristics, such as the rheological properties of exopolysaccharides (EPSs) used as stabilizers in the food industry [64,77], or the bio-preservative effects of LAB bacteriocins; (vii) they have a broad range of health-promoting effects; and (viii) they include antifungal and antibacterial agents [78] (Figure 2). Metabolomic techniques are widely used to evaluate the metabolic products of bacteria in feces and serum [79]. Many commercial pharmaceutical products, including cytoflora (components of *Lactobacillus* and *Bifidobacterium* sp.) and lacteol (inactivated *Lactobacillus* sps.), *Nyaditum resea* (inactivated mycobacteria), and others have already been approved for use as dietary supplements, immunomodulators, and instruments to lower the risk of infections [80]. Food ingredients such as immuno-biotics and post-immunobiotics can influence the immune response in two ways: augmentation or inhibition [81].

## 4. Production, Purification, and Characterization of Postbiotics

Billions of bacteria, containing more than three million genes, reside in the human gastrointestinal tract (GI), with the large intestine housing the most metabolically active and varied microbial community [82]. According to Tanaka and Nakayama [83,84], the human microbiota, which is specific to each individual, begins to appear in the uterus when the fetus consumes amniotic fluid and continues even after delivery. It is essential for human growth and contains more than 1000 different microbial species [27]. An individual’s gut microbiome contains both beneficial and harmful bacteria and reflects familial inheritance. The balance of both bacterial communities is essential; an imbalance disrupts this regular microflora (dysbiosis), which not only affects the GI tract but also has a severe effect on the operation of other organs. According to Carding et al. [85], this increases the risk of a wide range of infections and chronic disorders, including obesity, autism, psychological abnormalities, gastroenteritis, colon dysfunction, and irritable bowel syndrome. Several studies have demonstrated that this balance can be restored using postbiotics, which are less risky than prebiotics and probiotics [86]. Numerous studies have suggested that the beneficial effects of probiotics on human health are not always related to bacterial viability. A significant portion of the health advantages of pre-, pro-, and synbiotics appears to be mediated by different metabolic products, cellular and subcellular structural elements, and intact or ruptured dead microorganisms. Teichoic acid, short-chain fatty acids, vitamins, enzymes, exopolysaccharides, peptides, amino acids, and fermentation byproducts are among the postbiotics, which are structural and metabolic microbial products [16]. Probiotics produce these postbiotic components when they feed on prebiotics during lengthy storage or processing such as pasteurization, baking, or metabolic processes.

Numerous yeasts and bacteria have been utilized as probiotics; however, their postbiotic usage has not been explored. Postbiotic studies have recently attracted immense interest because postbiotics offer a secure substitute for live probiotics. Many functional foods, including the probiotic yeast *Saccharomyces cerevisiae* var. *Boular* and functional food products such as grains and fruit juices, have increased shelf stability, sensory characteristics, safety, and health benefits [87]. Modern technologies are used to extract, characterize, and examine the bioactivities of various postbiotic components in preparation for potential therapeutic applications in medicine [88]. Generally, postbiotics are stable and do not require cold chains for industrial use, whereas viable probiotic products must be stored and transported in cold storage facilities. There is no postbiotic interaction with the food matrix, no possibility of developing antibiotic-resistance genes, and no unacceptable taste or odor modifications. Therefore, postbiotics are a secure replacement for patients with immunological weaknesses following transplantation or in babies. Through the communication axes between the gut and target organs, such as the gut–brain, gut–lung, and gut–liver axes, postbiotics affect other organs, both locally and systemically [64].

The bacterial strain, culture medium, and how the bacteria are treated after they have multiplied are important factors that affect the type and quantity of postbiotic products. Food postbiotics are soluble components, such as commodities or metabolic byproducts, generated in a medium during bacterial growth and do not undergo any post-propagation processing [76]. Bacteria may occasionally be subjected to lysis after multiplication using cell fragmentation techniques, including thermal, enzymatic, chemical, sonication, hyperbaric, solvent extraction, or a combination of these [22,89]. These mechanisms add various extra intracellular metabolites and components made from the cell walls to the postbiotic mixture, enriching it and providing the ensuing postbiotics with new functions. The term “postbiotics” refers to a wide range of substances, such as extracellular vesicles (EVs), bacteriocins, enzymes, proteins, peptides, organic acids, vitamins, and other discharged compounds like EPS, cell wall components, polymers, teichoic acids, peptidoglycans, peptidoglycan-derived muropeptides, pili-type forms, cell surface fractions, cell-free extracts and lysates, culture supernatants, or biosurfactants and also the definition of postbiotics as described above [14,18,25,64,74,75,76,77]

**Figure 3:**
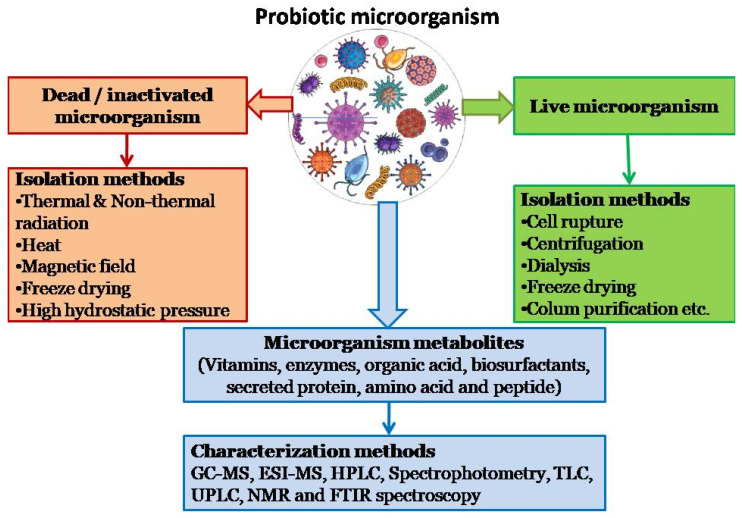
Isolation of postbiotics from probiotics and characterization methods.

Additional extraction and purification techniques such as centrifugation, dialysis, lyophilization, and column purification have been used on the resulting solutions to separate bacterial cells from postbiotic metabolites in both treated and untreated postbiotic mixtures [22,24]. The microorganisms used as starting points and the inactivation methods or techniques used for their production are generally used to characterize postbiotics because each process affects the quality and quantity of the final postbiotics produced and produces different postbiotics with different effects [27]. When prebiotics are consumed, stored, or processed for an extended period, such as during pasteurization or baking, or when metabolized, probiotics produce these postbiotic components. According to Aggarwal et al. [27], these postbiotics can be created using lab-utilizing techniques, including radiation (UV/ionizing), high pressure, high temperature, sonication, and formalin inactivation (Figure 3). Given the complexity of biological compounds with varying degrees of polymerization and glycosidic bonds, qualitative and quantitative analyses of postbiotics typically require complicated equipment and numerous concentration/purification steps using well-known techniques, such as chromatography, spectroscopy, NMR, Fourier transform infrared absorption spectroscopy, and spectrophotometry [19].

In food, complex microbial cultures in the intestine, or as a result of cell lysis, food-grade bacteria can emit postbiotics. Following the removal of the supernatant, the effects may be immediately examined or specific chemicals may be isolated for further study [16,20,22]. According to the analytical goals and type of characterization required, a suitable technique is commonly selected [19]. Various techniques have been developed and used to evaluate postbiotics qualitatively and quantitatively. Depending on the analytical objectives and qualitative and/or quantitative properties of microbial metabolite complexes, many analytical approaches are currently being used to identify postbiotic metabolites [22]. For the purpose of determining the qualitative and/or quantitative analysis and composition of postbiotics, a number of analytical techniques, including gas chromatography (GC), high-performance liquid chromatography (HPLC), thin-layer chromatography (TLC), and spectroscopic methods, have been reviewed in detail [68,90,91,92]. Owing to its high efficiency, resolution, sensitivity, accuracy, and low solvent usage, ultra-performance liquid chromatography offers superior postbiotic separation and identification capabilities [93,94,95]. Thin-layer chromatography has been used to determine whether postbiotics contain various compounds [96,97]. Metabolites in LAB postbiotics were quantified using colorimetric methods [98]. Lin et al.used nuclear magnetic resonance spectroscopy to understand the interactions between postbiotic biological metabolites [99,100]. Headspace solid-phase microextraction GC-MS was used to characterize sixty-two compounds in the volatile profiles of postbiotics from *Lactobacillus casei*. Additionally, the short-chain fatty acid content of the postbiotics from four different bacterial strains was studied using GC. HPLC is one of the most frequently used analytical techniques for postbiotic analyses, both quantitatively and qualitatively [101]. In a study by Li et al. [102], postbiotics from *Lactobacillus plantarum* were analyzed using the Fourier transform infrared spectroscopy method (FTIR) (Figure 3).

## 5. Classification of Postbiotics

The classification of postbiotics depends on various factors, including the type of microorganism, structural composition, and physiological functions. Various postbiotic compounds produced by extracellular and intracellular probiotic bacteria have also been identified. For example, muropeptides are derived from peptidoglycans, exopolysaccharides (EPSs), teichoic acids, surface-protruding molecules such as fimbriae, pili, or flagella that make up cell wall components, secreted proteins/peptides, bacteriocins such as acidophilin, reuterin, and bifidin, cell-free supernatants, organic acids, neurotransmitters, and biosurfactants [64,103]. Owing to their unique physical, chemical, and functional properties, postbiotics are classified into different types, including inactivated and dead probiotics, peptidoglycans, teichoic acids, exopolysaccharides, cell-free supernatants, short-chain fatty acids, bacteriocins, enzymes, and vitamins.

### 5.1. Inactivated and Dead Probiotics

Although other techniques, such as gamma or UV radiation, tyndallization, sonication, and chemical treatment, are used for the preparation of postbiotics, heat is the most frequently used method for the production of inactivated or dead probiotics. This inactivation process causes differences in the cellular makeup and biological functions [25]. According to studies conducted on experimental models, the biological characteristics of their viable counterparts, such as the ability to scavenge oxygen radicals, reduce inflammatory indicators, and modify host physiology, are retained by nonviable cells [104]. Recent studies on eight different strains of *Lactobacillus reuteri* suggest that both live and heat-killed cells of these bacteria adhered to caco 2 cell cultures and prevented enteropathogens such as *E. coli*, *Salmonella typhi*, *Listeria monocytogenes*, and *Enterococcus faecalis* from adhering to them [105].

### 5.2. Cell-Free Supernatants/Suspensions

Cell-free supernatants (CFSs) are a broad category of biomolecular and active metabolites with low or high molecular weights, such as organic acids, diacetylene, carbon dioxide, and bacteriocin-like substances, which are typically secreted by lactic acid bacteria and yeasts and may help maintain homeostasis in the body [106,107]. The composition of a medium can influence CFS composition. Cell-free supernatants (CFSs) are fluids that include nutrients from the growth medium that are not absorbed by microbes as well as any metabolites left over from microbial development. CFS, which is produced when microbes are fermented, has antibacterial, antibiofilm, anti-inflammatory, antioxidant, and anticancer activities and is used to treat diarrhea [108]. Generally, CFS is obtained from safe bacteria, and the bioactive material can be used as an alternative to common antimicrobials. The metabolites can be isolated from microbial cells by centrifugation and are highly abundant in anti-inflammatory, anticancer, antioxidant, phenolic, and flavonoid chemicals. These metabolites potentially increase the expression of anti-inflammatory cytokines like IL-10 and suppress pro-inflammatory cytokines like TNF α and IL-1β. Owing to the presence of organic acids, proteinaceous compounds, and fatty acids, the CFS generated by LAB may have an antimicrobial effect. Lactic and acetic acids, together with other substances, are principally responsible for the antibacterial action of LAB [109]. The anti-proliferative effects of a cell-free culture filtrate from *Lactobacillus fermentum* were also reported by Lee [110], who examined the anticancer capabilities of this substance. They used 3D spheroid cultures of colorectal cancer (CRC) cells as a model for their research. According to another study, cell-free *Lactobacillus reuterine* supernatant, which is likely to contain carbohydrates and fatty acid metabolites, has the potential to be used for the prevention and treatment of dental caries and periodontal diseases.

Hamad reported the antibacterial ability of culture suspensions produced from four probiotic strains, including *L. rhamnosus*, *L. fermentum*, *L. delbrueckii* subsp. *lactis*, and *Pediococcus acidilactici*, against *Clostridium perfringens* [111]. The growth of *Staphylococcus aureus*, *E. coli*, *Aspergillus niger*, and *Aspergillus flavus* is significantly suppressed by lactic acid, hydrogen peroxide, protein, and diacetyl generated from *Lactobacillus* and *Pediococcus* species culture filtrate [112]. The mechanism of inhibition appears to involve the creation of pores in cell membranes and cell lysis caused by lactic acid bacteria-producing bacteriocins, followed by the actions of diacetyl and bacteriocins. Lantibiotics (class I) are among the pore-forming peptides that are produced by lactic acid bacteria. These peptides typically form unstable pores and exhibit a wide range of activity. The majority of bacteriocins have interactions with anionic lipids, which are widely found in Gram-positive bacteria membranes. “Docking molecules” have the potential to improve the conductivity and stability of lantibiotic pores; “wedge-like” pores may be formed by antibiotics; and “carpet” or “barrel stave” pores may be formed by class II bacteriocins, which may increase membrane permeability [113].Hydrogen peroxide, fatty acids, secreted proteins, and organic acids have been detected in the culture suspension of the dental health probiotic *Weissella cibaria* strain CMU. Organic acids, secreted proteins, and hydrogen peroxide have all been shown to exert antibacterial activities against periodontal pathogens by disrupting cell membranes, lowering the pH of the cytosol, producing hydroxyl radicals, and interfering with cellular metabolic functions [94]. As with several biomolecules, CFS seems to have superior biological effects on host health compared with pure biomolecules [114]. Pyrrolo [1,2-a] pyrazine-1,4-dione has been observed in the CSF of several examined species of lactobacilli using GC-MS analysis. Strain-specific substances such as butyric acid, benzoic acid, biosurfactants (laurostearic acid), different peptides, fatty acids, ethanol, phenol, cyclopentanes, esters, and aldehydes are also present in strain-specific ways. Many of these substances exhibited antioxidant, biofilm removal, and antagonistic activities against *L. monocytogenes*, indicating their potential application as food additives, particularly *L. salivarius* [15]. The CFS antibacterial activity of Enterococcus faecalis was found to be thermostable and peaked at a neutral pH of 7.0, supporting its use in food preservation [115]. CFS is produced in various cultures, and bacterial strains exhibit differential functions. CFS derived from *L. acidophillus* and *L. casei* has antioxidant and anti-inflammatory effects [86]. *Lactobacillus* and *Bifidobacterium* also exert antibacterial activities by inhibiting *E. coli* strains [116]. It has been postulated that the antioxidant capacity of diverse intracellular fractions formed from *Lactobacillus* strains mediates an increase in cellular glutathione concentration, which is a significant non-enzymatic antioxidant essential for maintaining the intracellular redox state. However, these non-enzymatic postbiotic antioxidant properties may also have scavenging effects on ROS and reactive nitrogen species [26,117,118].

### 5.3. Cell Wall Fragments

The cell wall contains various components, including teichoic and lipoteichoic acids. Among the immunogenic components of bacterial cell walls, teichoic acids, lipoteichoic acids, and other compounds can elicit an immune response [119]. The cell wall of Gram-positive bacteria is mostly composed of lipoteichoic and teichoic acids, which account for approximately 60% of the cell wall mass [120]. Different lipoteichoic acid structures among the four strains of *Lactobacillus plantarum* lead to various immunological reactions in immune cells, as evidenced by the lipoteichoic acid recovered from the K8, K88, K5-5, and K55-5 strains of *L. plantarum* [121]. Teichoic acids are essential for the pathophysiology and development of antibiotic resistance [122]. According to Lebeer et al. [123], teichoic and lipoteichoic acids exhibit various bioactivities, including anticancer, immunomodulatory, and antioxidant activities.

### 5.4. Exopolysaccharides

According to Caggianiello et al. [124], lactobacilli and other bacteria produce a variety of homo- and heteropolysaccharides, including kefricin, glucans, and uronic acid. These are collectively referred to as exopolysaccharides (EPSs) and can be released extracellularly, cling to the surface of the microbial cell as a slime layer, or remain firmly attached as a capsule. These macromolecules have the power to defend against phages, phagocytes, and toxic substances; however, they also affect the immune system, physiological processes, lipid metabolism, and pathogen colonization in hosts. According to a study by Dinic et al. [125], EPS from *Lactobacillus paraplantarum* BGCG11 decreased proinflammatory (IL-I, TNF, and iNOS) and concurrently elevated anti-inflammatory (IL-6 and IL-10) cytokines, thereby reducing inflammation in rats. According to Liu et al. and Wang et al. [74,126], EPSs generated from probiotic *Lactobacillus fermentum* and *Paenibacillus polymyxa* cultures show antioxidant activity and may thus have therapeutic effects in diseases such as diabetes, atherosclerosis, and rheumatoid arthritis. Additionally, EPSs extracted from pathogenic *E. coli* and *S. aureus* prevented the development of biofilms and suppressed tumor growth and inflammation [74]. The bioremediation, pharmaceutical, food, and textile industries have significant applications of EPSs derived from various bacteria [127]. Examples of food additives include xanthan, alginate, gellen, levans, and pullulan [128]. Centrifugation is the first stage in a multiphase method to extract EPSs, which also includes acid protein removal, cold ethanol precipitation, filtration to remove small molecules, dialysis, and lyophilization [129]. EPSs are essential for cell adhesion and defense, and the structural diversity of EPSs produced by lactic acid bacteria (LAB) enables polymers to have a range of bioactivities, including immunomodulatory, antitumor, antimutagenicity, antioxidant, anti-inflammatory, antihypertensive, antibacterial, antiviral, cholesterol-lowering, and anti-gastrointestinal activities [130]. Khalil et al. [131] reported that EPS generated from *Lactobacillus* strains showed antibacterial and antioxidant activities and improved lipid metabolism by inhibiting cholesterol absorption. By increasing the activities of antioxidant enzymes, such as catalase, glutathione peroxidase, and superoxide dismutase, and lowering the levels of lipid peroxidation in serum and mouse livers, EPSs generated from *Lactococcus lactis* subsp. *lactis* displayed antioxidant activity [132]. Currently, the food industry uses EPSs as emulsifiers, stabilizers, and water-binding agents.

### 5.5. Enzymes

Enzymes are proteins that catalyze biological reactions. Based on their activities or functions, enzymes can be classified into six primary groups: oxidoreductases, transferases, hydrolases, lyases, isomerases, and ligases [133,134]. A small number of bacterial strains, primarily *Bacillus subtilis* and *Bacillus licheniformis*, as well as a few fungal strains, notably, *Aspergillus niger* and *Aspergillus oryzae*, are the primary sources of enzymes that are used in a variety of physiological, metabolic, and regulatory processes. A significant amount of glutathione peroxidase was detected in two strains of *Lactobacillus fermentum*, which was later discovered to possess strong in vitro antioxidant capabilities. Under difficult conditions such as temperature, pH, organic solvents, oxidizing agents, and detergents, *Bacillus* spp.can produce proteolytic enzymes in large yields that are remarkably stable. Catalase from a genetically modified strain of *L. lactis* can protect mice from chemically induced colon cancer [135,136,137].

### 5.6. Short Chain Fatty Acids (SCFAs)

Short-chain fatty acids (SCFAs) are an important class of compounds produced by gut bacteria such as Bacteroides and Firmicutes, which ferment plant polysaccharides [138]. Inulin and fructooligosaccharides, two prebiotics, are fermented to produce SCFAs, primarily acetate, propionate, and butyrate, which are found in the colon and feces at an estimated 60:20:20 molar ratio and aid in the regeneration of the intestinal epithelium [139,140]. In addition, they suppress the production of pro-inflammatory cytokines, preventing the activation of nuclear factor-kappa B (NF-κB). A reduction in atherogenesis in a mouse model was demonstrated using an in vivo butyrate model [141]. Acetate and lactate are produced by bifidobacteria when too many carbon atoms are available for development. Inhibiting the growth of *Klebsiella oxytoca*, for instance, *Lactobacillus acidophilus*, *Lactobacillus fermentum*, *Lactobacillus paracasei* ATCC 335, and *Lactobacillus brevis* produced SCFAs by lysing the cell wall [142]. SCFs exert several beneficial effects on health. In addition to improving colonic function and lowering pH, they promote the proliferation of epithelial cells and blood flow in the colon [143]. Bird et al. [144] found that SCFAs significantly lowered the prevalence of colorectal diseases. When colonic bacteria ferment undigested carbohydrates, they produce mostly acetate, propionate, and butyrate in ratios that normally vary from 3:1:1 to 10:2:1. Acetate aids cholesterol regulation and is used as a growth factor by other bacteria. Propionate and butyrate play a role in gluconeogenesis, providing colonocytes and epithelial cells with their main source of energy and promoting apoptosis of colon cancer cells [145].

### 5.7. Bacteriocins

Lactic acid bacteria (LAB), as well as other eubacteria and archaebacteria, produce tiny ribosomally synthesized peptides or proteins known as bacteriocins that can either kill or impede the growth of other bacteria. According to Soltani et al. [146], the therapeutic utility of bacteriocins as next-generation antimicrobials for reducing the threat posed by drug-resistant pathogenic organisms is highlighted by their restricted broad-spectrum inhibitory effect against bacterial growth. Examples include nisin, subtilosin, lactococcin G&Q, enterocin, lactocyclicin, bovicin, plantaricin, and lacticin, among others [147]. Bacteriocins have demonstrated potential for use in food preservation. Nisin was the first bacteriocin to receive regulatory approval for commercial use as a food preservative from organizations such as the European Food Safety Authority (EFSA), Food and FDA, and Health Canada. Currently, it is used as a food additive in more than 80 countries. Bacteriocins prevent pathogen growth in the GI tract by creating pores in cell membranes, preventing the proper construction of cell walls, and inhibiting enzyme and protein functions. Multibacteriocinogenic strains of *L. paracasei* and *L. taiwanensis* show antibacterial activity against *E. coli*, *Salmonella gallinarum*, and enteropathogenic *E. coli* [148,149]. Because of their various qualities, bacteriocins have been widely used in various applications, including medicine, cancer therapy, food, cosmetics, and veterinary medicine.

### 5.8. Vitamins

Vitamins are thermosensitive chemical substances that are necessary for the body to perform a number of physiological processes, including DNA replication, repair, and methylation, and vitamins must be supplied exogenously.Vitamins play a crucial role in many physiological processes such as bone health, brain function, and blood clotting, and riboflavin acts as a hydrogen carrier in redox reactions. Vitamin K also plays a role as a cofactor of gamma carboxylase activity in blood clotting, and various other critical vitamins, such as vitamin K, and various B-group vitamins, such as folate, riboflavin, cobalamin, pyridoxine, thymine, niacin, and nicotinic acid, are produced by lactic acid bacteria and *Bifidobacterium* sp. [150]. Numerous fermented foods, including fermented milk, yogurt, and cheese, are major sources of these vitamins, which help the digestive system. In addition to being essential for producing energy, controlling genes, and changing intestinal immunity, B-group vitamins, including B12, B2, B6, B9, and vitamin K, may all be synthesized by the gut microbiome on their own. For example, vitamins B2, B6, and B9 exerted anti-tumorigenic effects against pro-monocytic lymphoma cells [151].Cobalamin, generally known as vitamin B12 (B12), is a water-soluble vitamin essential for maintaining hematopoiesis and neuronal health. It is also an essential nutrient in animal products. Probiotics such as *L. sanfranciscensis*, *L. reuteri*, *L. rossiae*, and *L. fermentum*, which have been shown to synthesize vitamin B12 and could be useful substitutes for industrial production, have recently been found to contain genes encoding enzymes necessary for cobalamin (B12) synthesis [152,153,154]. In contrast to MK-6, MK-8, and MK-9, which are produced by *Bacteroides fragilis*, MK-10, MK-11, and MK-12 are produced by *Eubacterium lentum*, *Lactococcus lactis* ssp. *lactis*, and *Lactococcus lactiscremoris* [155]. Cortés-Martin et al. [156] found that the gut microbiota also produces dietary polyphenols. Aromatic amino acids are generated and metabolized in the gut to function as bioactive molecules in circulatory, renal, and brain systems [157].

### 5.9. Neurotransmitters

Neurotransmitters, such as serotonin, dopamine, norepinephrine, catecholamines, and acetylcholines, are produced by gut bacteria such as *Bifidobacterium*, *Lactobacillus plantarum*, *Lactobacillus brevis*, and *Bacillus subtilis*. These neurotransmitters play a major role in brain function via the gut–brain axis through the modulation of enteric nerve signaling. Tryptophan is an amino acid that is transformed into serotonin, which is responsible for mood improvement. Gamma-aminobutyric acid inhibits neurotransmission, and when it does not work, anxiety and depression result. Acetylcholine and catecholamines are essential for CNS activities, such as emotion, memory, learning, and motor control [157,158,159]. According to Patterson et al. [159], microbiome management can cure mental conditions linked to depression, and these compounds appear to have antidepressant potential.

### 5.10. Extracellular Vesicles

EVs are spherical, lipid bilayer, membrane-bound particles that release commensal bacteria, such as *E. coli* and *Akkermansia muciniphila*, into the environment. They are involved in the horizontal transfer of genetic material across bacterial species and contain a variety of substances, including proteins, DNA, RNA, glycolipids, polysaccharides, enzymes, and toxins. According to studies by Ahmadi Badi et al. and Chelakkot et al. [159,160], these substances are thought to regulate the permeability of the gut barrier and signaling pathways, maintain intestinal homeostasis, improve lipid profiles, and facilitate communication between the gut and brain. Survival, competitiveness, pathogenesis, and immunomodulation are some mechanisms regulated by bacterial EVs. They can also swiftly cross the mucosal barrier and interact with the host, thereby lowering the risk of sepsis. Previous studies have shown an association between obesity and reduced barrier integrity. Increased intestinal barrier permeability causes metabolic endotoxemia, which is the primary contributing factor to obesity-related metabolic diseases [161,162,163]. EVs derived from *Akkermansia muciniphila* reduced fat accumulation, body weight gain, and pathological abnormalities in high-fat diet (HFD)-fed mice; the tested EVs had the most significant effects on adipocyte size, epididymal white adipose tissue (eWAT) weight, lipid balance, and expression of inflammatory cytokines in the adipose tissue and glucose tolerance in diabetic mice. EVs derived from *Propionibacterium freudenreichii* can mitigate inflammation by modulating the NF-B pathway [161,164,165,166,167].Recently, Gurunathan et al. reported that *Pseudomonas aeruginosa*-derived outer membrane vesicles exhibited antibacterial and antibiofilm effects against *Streptococcus mutans.* Extracellular nanovesicles produced by *Bacillus licheniformis* showed anticancer effects against breast and lung cancer cells [167].

## 6. Applications of Postbiotics in the Food Industry

Functional foods, such as probiotics, prebiotics, and postbiotics, have recently received considerable attention from researchers, manufacturers, and consumers. The development of innovative functional foods and preventive medicine formulations for improving host health, as well as accurately characterizing their mechanisms of action, is currently the focus of a sizable section of postbiotic research [168]. A variety of food products with bioactive ingredients, such as probiotics, dairy, and non-dairy products, are already available in the market to meet the nutritional needs of consumers with various dietary preferences, such as those who are allergic to milk proteins, lactose intolerant, and vegetarians [168,169]. Source, components, types, and functional aspects of postbiotics is shown in Table 1.

Postbiotics are stable over a wide range of temperatures and pH levels, making it easy to add meals and components before thermal processing without affecting their effectiveness. Producers may gain from this, both technically and financially. They can be used in delivery systems such as pharmaceutical goods and/or functional meals because the right amount of postbiotics can be managed under manufacturing and storage conditions when survival is not the main determining factor [168]. Bacterial lysates containing cell surface proteins, enzymes, peptides, metabolites, neuropeptides, and lower organic acids such as lactic acid are examples of postbiotics. Fermentation is the most common postbiotic source in the food industry. Postbiotics are naturally present in many dairy products, such as kefir, kombucha, yogurt, and pickled vegetables. Generally, *Lactobacillus* sps., *Bifidobacterium*, *Saccharomyces*, *Bacillus*, *Streptococcus*, or *Faecalibacterium genera* are highly effective postbiotic-producing microorganisms in the form of cytoplasmic extracts and cell wall components [33,73,206,207].

According to several studies [28,30,34,72,208], several *Lactobacillus* species, including *L. rhamnosus*, *L. bulgaricus*, *L. acidophilus*, *L. reuteri*, *L. casei*, and *L. fermentum*, play significant roles in the food industry. On the other hand, the most prevalent probiotic producers of postbiotics are *Bifidobacterium* sps., including *B. bifidum*, *B. longum*, *B. breve*, and *B. longum*. According to an in vivo study, mice injected with *B. longum* showed a strong antibody response; however, the cell wall and cytoplasmic fractions had little effect on the immune system [209]. Additionally, *L. plantarum* species are thought to be potential food bio-preservatives and benefit animal gut health because they produce metabolites with high levels of mixed organic acids and bacteriocins. Nisin, a bacteriocin produced by *Lactococcus lactis* subsp. *lactis*, is used as a preservative in many foods including dairy products, infant formula, and canned soups [210]. EPS produced by LAB, such as *L. rhamnosus*, which are important in dairy products, may enhance the physicochemical and sensory qualities of food-based products [20]. Owing to the postbiotic composition of the supernatant from *Lactobacillus plantarum* YML007, the shelf life of soybeans was extended by up to two months [211]. Postbiotic enzymes, including purified phytases from *Bifidobacterium pseudocatenulatum* and *Bifidobacterium longum* spp. *infantis*, increase the amount of myoinositol triphosphate while lowering the amount of phytate in cereal combinations [212].

Cereals lose some vitamin B content when heated or ground. Vitamins B1, B2, B3, B9, B11, and B12 can be produced by additional bacteria as a result of grain fermentation and LAB pretreatment, which can compensate for these vitamin losses. Cereals that underwent LAB fermentation had considerably higher levels of total lysine, protein fractions, sugars, and soluble dietary fiber and a higher bioavailability of calcium, iron, and zinc. Wheat may also produce antioxidant peptides, γ-aminobutyric acid, and angiotensin I-converting enzyme-inhibitory peptides via LAB fermentation [213,214,215].

Postbiotics are chemicals obtained from specific bacteria that prevent microbiological food degradation and increase the shelf life of food. Postbiotics are highly significant in the food industry because they exhibit anti-microbial activity against both pathogenic and spoilage microorganisms through a variety of mechanisms, such as creating cavities in CM, changing proteins in cell walls, and lowering the pH of the bacterial cytoplasm [216]. The nutritional value and organoleptic changes in non-vegetarian food can be preserved by directly applying a postbiotic coating (for example, to fish fillets and slices of meat) or by spraying it (for example, on ground fish and meat), depending on the type of meat and postbiotic. Postbiotics comprising *Pediococcus acidilactici*, *Latilactobacillus sakei*, and *Staphylococcus xylosus* flavonoids and phenolics have been shown to reduce Salmonella typhimurium in chicken drumsticks [217]. By consuming products made from the fermentation of *Saccharomyces cerevisiae*, it may be possible to limit the amount of Salmonella enterica contamination in poultry products by consuming products made from *S. cerevisiae* fermentation [218]. Postbiotic-containing preservatives were found to be as effective as frequently used commercial preservatives in preserving vacuum-packaged cooked sausages as natural preservation technologies [219]. Postbiotics can be used as an alternative biocontrol for the safe production of dairy products, fruits, and vegetables. For instance, several bacteriocins and LABs play roles in controlling cheese-blowing errors. Postbiotics can be used as sanitizers in the food industry [15].

## 7. Overall Therapeutic Effects of Postbiotics

Postbiotics typically contain additional therapeutic and health-promoting components. Metabolites sustain healthy bacteria and reduce the chances of harmful living microorganisms [220]. Numerous bacteria in the gastrointestinal system catabolize indigestible carbohydrates to produce large amounts of butyrate and volatile SCFAs [221]. Enzymes that protect cells against oxidative damage, cancer, or heart disease, recognized as immunostimulants by teichoic acids, peptide antimicrobials that directly combat invasive bacteria in the colon, and the prebiotic/indigestible carbohydrate inulin have been shown to reduce constipation and increase the volume of feces. Postbiotics reduce lipid levels, inhibit fatty acid production, and prevent inflammatory diseases [220,222]. Digestible oligosaccharides enhance calcium absorption in humans, particularly during puberty and menstruation. The amounts of calcium, potassium, and magnesium ions in the intestinal lumen increase when cellular transformation is controlled and prevented in the intestinal lumen [20]. Postbiotics rich in butyrate and oligosaccharides are important for cancer prevention. Because of the presence of bacteriocins and organic acid-based postbiotics, *Lactobacillus acidophilus* LA5, *Lactobacillus salivarius*, and *Lactobacillus casei* 431 displayed antibiofilm effects against *L. monocytogenes*. Therefore, postbiotics can be used in the food industry to prevent and reduce bacterial biofilm development [16,223].Reactive oxygen species (ROS) can change the nature of lipids and proteins and eventually lead to cellular dysfunction, which can lead to permanent malfunctions such as diabetes and its consequences, microvascular disease, and cardiovascular effects. Living organisms can use enzymatic or non-enzymatic defenses, such as natural antioxidants (vitamins C and E), to quench ROS [224,225]. Superoxide dismutase (SOD), an antioxidant enzyme that aids in reducing free radical accumulation in the intestinal and colon lumen of rats, was found to be active in the cell-free extracts of strains belonging to *Lactococcus* and *S. thermophilus*, with *Lactococcus* exhibiting more activity than *S. thermophilus* [26].The *Lactococcus lactis* strain produces catalase (CAT), which is able to increase CAT activity in mice administered 1,2-dimethylhydrazine (DMH), which improves antioxidant capacity by decreasing H_2_O_2_ levels and preventing or reducing the severity of intestinal diseases caused by ROS [137]. Several lactic acid bacteria, including *Bifidobacterium adolescentis*, *B. longum*, *B. infantis*, and *B. breve*, can break down hydrogen peroxide. *L. plantarum* has been shown to have a significant antioxidant role through an increase in GPx concentration in serum and ruminal fluid of post-weaning lambs.Among the 25 distinct lactobacilli, the human strain *L. plantarum* 30 B has the highest catalase activity, whereas the human strain *L. acidophilus* 900 has the highest superoxide anion dismutation activity [67,226,227,228]. Additionally, in an animal model of inflammatory bowel disorder (IBD), *L. acidophilus* strain 900 suppressed the inflammatory process more effectively than *L. plantarum* strain 30 B, indicating that H_2_O_2_ is less harmful than superoxide anion radicals and ROS [227]. Collectively, the different Lactobacillus strains exhibit anti-inflammatory potential by expressing various antioxidant enzymes. *Lactobacillus fermentum* E-3 and E-18 were isolated from the intestinal microflora of a healthy child and displayed antioxidant activity by expressing high levels of GPx and MnSOD in the intestine, which are crucial for preventing lipid peroxidation and removing hydrogen peroxide [228].

Any drug or bioactive substance that suppresses inflammation may potentially have anticancer properties because inflammation and carcinogenesis are closely associated. Probiotics from fermented milk by *Propionibacterium freudenreichii* induce apoptosis in HGT-1 human gastric cancer cells [229]. The supernatant of *L. rhamnosus* GG lowered the expression of MMP-9, which aided in the breakdown of the intercellular matrix, promoted cancer cell penetration, and increased ZO-1 expression [230]. *Lactobacillus plantarum*-derived postbiotics exhibited selective cytotoxicity and increased apoptosis against MCF-7 and RG14 PM on HT29, RG11, and RI11 cells [231]. The severity of 2,4,6-trinitrobenzenesulphonic acid (TNBS)-induced colitis in mice was reduced with the oral administration of *Faecalibacterium prausnitzii* supernatant, and dysbiosis related to TNBS colitis was similarly improved [33]. An anti-inflammatory immunogen derived from yeast culture was shown by Jensen et al. [73] to activate human natural killer cells and B lymphocytes as well as to change the expression of chemokine receptors. According to Jensen et al. [73], cell wall fragments and metabolites from *Bacillus coagulans* cause human polymorphonuclear (PMN) cells to spontaneously inhibit and produce reactive oxygen species (ROS) in response to oxidative stress.Interleukin-12 (IL-12) production is inhibited and interferon-gamma (IFN-) and tumor necrosis factor-alpha (TNF-) production is stimulated by a peptidoglycan generated by *Lactobacillus* spp. However, peptidoglycans can also promote the synthesis of pro-inflammatory cytokines in macrophages, such as TNF- or IL-12, as well as the expression of IL-12p35 mRNA [232].

Levan, a polysaccharide derived from *Bacillus licheniformis*, was found to be efficient in preventing hyperglycemia and oxidative stress induced by diabetes in adult rats, suggesting that adding levan to the diet may help prevent diabetes-related molecular abnormalities such as blood glucose levels, improvement in peripheral sensitivity to residual insulin, and activation of Langerhans islets [233]. In experimentally induced diabetes mellitus (DM), folic acid administration lowers glycemia and improves the activity of specific enzymes, including superoxide dismutase (SOD) and catalase (CAT). Folic acid functions as an antioxidant by lowering the production of superoxide radicals catalyzed by nitric oxide synthase (NOS) [234].EPSs from *Bacillus subtilis* sp. *suppress* (BSEPS) control hyperglycemia and dyslipidemia in diabetic rats by increasing insulin levels and decreasing blood glucose and troponin blood concentrations [235].The fact that EPSs from *Lactobacillus plantarum* H31-2 decreased the amount of glucose in the supernatant of insulin-resistant HepG2 cells suggests that EPS H31-2 may help these cells to take up glucose through the AMPK/PI3K/Akt pathway. The expression of the glycometabolism-related genes glucose transporter 4 (GLUT-4), protein kinase B (Akt-2), and AMP-activated kinase (AMPK) was also elevated by EPS H31-2. According to these findings, *Lactobacillus plantarum* EPS H31-2 may effectively inhibit pancreatic amylase activity, which lowers blood glucose levels in type 2 diabetes (T2DM) patients, indicating that it may be utilized to both prevent and treat diabetes [236].In a high-fat diet and streptozotocin-induced type 2 diabetes in mice, a mixture of multiple *Lactobacillus* species reduced fasting blood glucose (FBG), hemoglobin A1c (HbA1C), and leptin levels and improved glucagon-like peptide-1 (GLP-1) levels [237]. In the absence of detectable changes in the composition of the microbiota or metabolome, *L. plantarum* bacteriocin plantaricin effectively reduced body weight and food intake in mice fed a high-fat diet (HFD) [238]. By activating NOD2, peptidoglycan-containing muramyl dipeptide (MDP) was demonstrated to be an insulin-sensitizing postbiotic that can reduce adipose tissue inflammation and glucose intolerance in obese mice without influencing body weight or altering microbiota composition [239]. NOD2 protects against inflammatory colitis by reducing insulin resistance and inflammation induced by other bacterial products. NF-ҡB and receptor-interacting serine/threonine-protein kinase 2 (RIPK2) are both involved in NOD2 activation [240,241,242]. A preclinical model of *Caenorhabditis elegans* suggested that *Bifidobacterium animalis* subsp. *lactis* BPL1-derived lipoteichoic acids (LTAs) function as novel lipid modulators with fat-reducing capabilities through the insulin-like signaling pathway (IGF-1) and prevent metabolic syndrome and diabetes-related disorders [243].

According to Taverniti and Guglielmetti [25], the postbiotic produced by lactic acid bacteria can quickly interact with immune cells and the epithelium to activate innate immunity. For instance, *Lacticaseibacillus rhamnosus* HN001 improves leukocyte phagocytic activity, which boosts immunity, and postbiotics produced by *L. gasseri* TMC0356 have immunomodulatory activity [244], and these bacteria carry out their immunomodulatory activities by increasing Th1-associated cytokine levels and decreasing Th2-related cytokines [245]. One study found that the LPS-induced TLR-4 pathway improved the ability of peptidoglycans from several *Lactobacillus* species to suppress the production of inflammatory cytokines in macrophage-like cell types [246]. Conversely, a combination of heat-inactivated probiotic strains, including *Lactobacillus* sps., protected intestinal cells from infection by *E. coli* in in vitro models of the intestinal mucosa (HT29-MTX cells), ensuring the restoration of tight junction function and membrane integrity, preventing an increase in paracellular permeability and penetration of pathogens into the intestinal epithelium, and modulating cytokine gene expression. Another study found that the probiotic strain *Streptococcus thermophilus* CRL1190 and its EPSs decreased *Helicobacter pylori* adhesion and lowered the inflammatory response in a human gastric adenocarcinoma epithelial cell line (AGS cells) [247]. Heat-killed *S. boulardii* can maintain the gut barrier by preserving intestinal permeability at physiological levels, lowering bacterial translocation, and preventing mucosal lesions; thus, heat-killed *S. boulardii* treatment can maintain the gut barrier [248]. Similarly, another study found that the metabolic byproducts of an infant formula fermented with *Lactobacillus paracasei* CBA L74 can protect the host from pathogens and enteric pathogens by inhibiting immune cell inflammation, and these byproducts also have protective effects against colitis [249]. When administered orally to newborn rats infected with *E. coli* K1, a new secretory protein, HM0539, produced by *Lacticaseibacillus rhamnosus* GG, was used to prevent and treat diseases related to intestinal barrier dysfunction. HM0539 promoted the development of neonatal intestinal defense and was sufficient to prevent *E. coli* K1 pathogenesis. The researchers also discovered that HM0539 protected against liver damage, colitis induced by dextran sulfate sodium (DSS), and bacterial translocation induced by LPS/D-galactosamine [250].Postbiotic *Lactobacillus casei* Zhang (LcZ) increases the production of proinflammatory cytokines and the transcription of TLR2, TLR3, TLR4, and TLR9, boosting the macrophage-mediated innate immune response [251]. LcZ was heat-inactivated and resuspended at a concentration of 10^6^ CFU/mL in PBS. The inactive preparation boosted the secretion of cellular immune markers more than the live preparation, according to a study using the live and inactive forms of *Bacillus amyloliquefaciens* FPTB16 and *Bacillus subtilis* FPTB13 [252]. In addition, a mouse study found that combining heat-inactivated LAB with two heat treatments—30 min at 100 °C and 15 min at 121 °C—increased macrophage immunomodulatory activity [253]. Compared with heat-inactivated postbiotics from *Enterococcus gallinarum* L-1, ultraviolet-inactivated postbiotics were more effective in boosting phagocyte function. The results showed that the postbiotic *Lactobacillus gasseri* TMC0356 increased IL-12 production in macrophages more compared toprobiotics. This suggests that heat treatment enhances the capacity of the strain to activate IL-12 production in macrophages and that the postbiotic form has a greater immunomodulatory effect than the probiotic form. Postbiotics produced by *Lactobacillus acidophilus A2*, *Lactobacillus gasseri A5*,and *Lactobacillus salivarius A6* alter the Th1-mediated immune response by promoting the proliferation of IL-10 and IL-12 p70, IFN-production in splenocytes, and IL-12 p70 secretion in dendritic cells, respectively [24,252,253]. SCFAs potentially inhibit the production and function of regulatory T cells by inhibiting histone deacetylase (HDAC) induction and are also important factors in building the link between the microbiome and the immune system [254]. *Lactobacillus acidophilus* and *Lactobacillus casei* have been shown to increase interleukin-10 (IL-10) production while simultaneously decreasing pro-inflammatory tumor necrosis factor (TNF-) cytokine secretion [86]. It has been demonstrated that soluble components derived from *L. reuteri* strain CRL1098 significantly reduce the release of pro-inflammatory mediators such as nitric oxide (NO), cyclooxygenase 2 (COX-2), heat shock proteins 70 (Hsp70), TNF-, and IL-6 [255]. Similar anti-inflammatory effects were demonstrated by *S. boulardii*, which were attributed to excrete low-molecular-weight-soluble components that play a role in inhibiting NF-ҡB activation and NF-B-mediated IL-8 gene production in monocytes and intestinal epithelial cells [256].

## 8. Antiviral, Antibacterial, Antioxidants, Anticancer, and Anti-Inflammatory Mechanisms of Postbiotics

### 8.1. Antiviral

**Figure 4:**
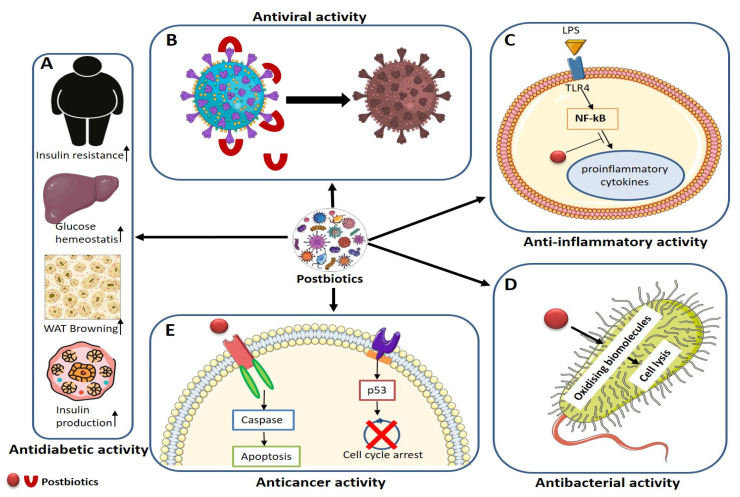
A scheme illustrating the possible mechanism and application of antidiabetic, antiviral, anti-inflammatory, antibacterial, and anticancer effects of postbiotics. (**A**) Postbiotics increase antidiabetic activity by increasing insulin production, WAT Browning, glucose homeostasis, and insulin resistance. (**B**) Postbiotics inhibit viral activity by blocking the receptor-binding site of a virus in the host system. (**C**) Anti-inflammatory activity by suppressing the production of proinflammatory cytokines. (**D**) Antibacterial activity by oxidizing biomolecules and forming pores in the membrane. (**E**) Anticancer activity by caspase activation, cell cycle arrest, and apoptosis.

Viral diseases pose significant global public health hazards, underscoring the importance of antiviral medications and vaccines in preventing infections. Postbiotic antiviral effects predominantly manifest through the restraint of viral attachment to host cells, thereby delaying the onset of infection (Figure 4). Postbiotics inhibit viral binding to host cell receptors, preventing viral entry and fortifying the host immune system [257,258].

The immune system plays a pivotal role in actively safeguarding cells, eliminating viruses, and initiating a pro-inflammatory response to establish Th1-type immunity. This involves the production of inflammatory chemokines, cytokines, and interleukins, including TNF, interferons, IL-23, IL-18, and IL12. Additionally, T-lymphocytes, NK cells, and monocytes/macrophages are activated to produce cytokines [259].

Probiotics and their metabolites protect against viral infections by enhancing both innate and adaptive immunity. This results in a reduction in sickness duration, virus shedding, and the frequency of episodes. Furthermore, postbiotics normalize intestinal permeability and augment the production of virus-specific antibodies [260].

Postbiotics exhibit antiviral efficacy by impeding retroviral reverse transcriptases and preventing viral uptake by host cells. The specific probiotic strain used and the type of virus significantly influence the antiviral effects of postbiotics. For instance, Anwar et al. [261] demonstrated that plant-derived probiotics, known as plantaricins, mitigate SARS-CoV-2 infection by modulating the immune system. Plantaricin compounds inhibit COVID-19 pathogenicity by binding to the spike glycoprotein (S) [262]. Metabolites from *L. rhamnosus* deter viruses from attaching to cell lines [263].

The organic acids of probiotic bacteria, particularly those produced by attaching to the glycoprotein (S) of viruses, exhibit an antiviral mechanism, preventing viruses from attaching to the angiotensin-converting enzyme (ACE2) [264]. Lauric acid and meristic acid are reported to significantly inhibit the growth and development of viruses [265]. Furthermore, organic acids produced by probiotics bind to the spike glycoprotein (S), preventing it from interacting with the angiotensin-converting enzyme 2 (ACE2). Microbial-derived peptides hinder viral proliferation by inhibiting endosomal acidification [266].

### 8.2. Antibacterial

The primary bioactive components of postbiotics consist of organic acids, including lactic acid, acetic acid, tartaric acid, malic acid, and citric acid. These acids inhibit bacterial growth by reducing pH levels and altering membrane integrity. Bacteriocins contribute to inhibiting bacterial viability by targeting bacterial cytoplasmic membranes and generating spores [168,267,268,269,270].

Short-chain fatty acids, such as lauric and meristic acids, effectively inhibit bacterial formation and proliferation. These acids disrupt the electron transport chain, alter the structure and activity of enzymes, and induce morphological and functional changes in delicate components like proteins. As a result, short-chain fatty acids cause cell lysis and enhance membrane permeability in bacteria [271]. Recently, Gurunathan et al. reported that *Pseudomonas aeruginosa*-derived outer membrane vesicles exhibited antibacterial and antibiofilm effects against *Streptococcus mutans* [167]. Antibacterial peptides produced by bacteria exhibit robust antibacterial activity by rupturing microbial membranes and accumulating metabolic nuclei. The mechanisms underlying the antibacterial action of peptides involve creating physical holes that facilitate the leakage of cellular content, causing damage to delicate microbial intracellular components, initiating lethal processes such as inducing hydrolases that negatively impact cell walls, and acidifying the bacterial cell membrane (Figure 4) [168,272,273,274].

### 8.3. Anticancer

Postbiotics exhibit anticancer properties by inhibiting cell growth and proliferation and enhancing apoptotic effects. Polysaccharides, among the various components of postbiotics, including SCFAs, metabolites, microbial cell fractions, functional proteins, EPSs, and cell lysates, demonstrate the most prominent anticancer activity [13,275]. The molecular mechanisms underlying postbiotic anticancer effects involve the modulation of immune responses, suppression of mutagenesis and carcinogens, activation of pro-apoptotic pathways, decreased bacterial translocation, and increased apoptosis and autophagy for the prevention and treatment of cancer (Figure 4).

Numerous in vitro and in vivo experiments have elucidated the anticancer properties of postbiotics. For instance, SCFAs from propionibacteria inhibit human colorectal cancer cell lines HT-29 and Caco-2 by inducing the loss of mitochondrial transmembrane potential, generating reactive oxygen species, activating caspase-3, modulating Bcl-2 regulation, and inducing nuclear chromatin condensation [276]. The administration of the cytoplasmic fraction of lactic acid bacteria (LAB) enhances specific antitumor activity by modulating cellular immunity [277]. *Lactobacillus salivarius* REN probiotics inhibit oral carcinogenesis in a dose-dependent manner, providing defense against oxidative damage, reducing COX-2 and PCNA expression, and protecting against oxidative damage [278]. A FACS analysis of human colorectal cancer RKO cells revealed that SCFAs generated by P. freudenreichii increase the sub-G1 phase and decrease the S and G2/M phases [279].

In human colorectal cancer cells (T4056 and HT-29), cell-free *L.* spp. pentasaccharides induce apoptosis and prevent the progression of the S-phase cell cycle. In human colorectal SW480 cancer cells, cytoplasmic extracts and cell walls of *L. lactis* spp. demonstrate an antiproliferative effect linked to a lower expression of cyclin D1 [280]. The probiotic-derived p8 protein exhibits antiproliferative activity by inhibiting the p53-p21-Cyclin B1/Cdk1 signal pathway, leading to growth arrest at the G2 phase of the cell cycle in human colorectal DLD-1 cells [281]. Exopolysaccharides derived from *L. acidophilus* 606 inhibit the proliferation of HT-29 colon cancer cells by directly affecting cell morphology and activating autophagic cell death through the induction of Bcl-2, Bak, Beclin-1, and GRP78 [282].

Postbiotics eliminate tumor cells by modulating immune responses and activate anti-inflammatory and pro-apoptotic cytokines such as TRAIL, interleukin (IL)-10, and TGF-β [283]. SCFAs protect the mucosal layer by lowering the levels of immunomodulators such as prostaglandins in human breast cancer MCF7 cells [284]. Heat-killed *L. pentosus* b240 promotes the production of immunoglobulin A (IgA), IL-6, IL-10, interferon (IFN)-γ, and tumor necrosis factor, but not IL-4, IL-5, B-cell activating factors, IFN-α, IFN-β, and transforming growth factor-β1 [285]. *L. plantarum* strain YYC-3 strongly inhibits human colorectal cancer HT-29 and Caco2 cell lines by modulating the immune system, downregulating the expression of inflammatory cytokines interleukin IL-6, IL-17, and IL-22, and reducing the infiltration of inflammatory cells [286]. Extracellular nanovesicles produced by *Bacillus licheniformis* showed anticancer effects against breast and lung cancer cells [193].These studies collectively indicate that postbiotics possess anticancer properties.

### 8.4. Anti-Diabetic

Diabetes mellitus is a chronic, irreversible condition characterized by dysregulated insulin response or synthesis in the body. Type 1 diabetes mellitus (T1DM) and type 2 diabetes mellitus (T2DM) represent distinct forms of diabetes. T2DM, accounting for approximately 90% of diabetes cases, arises from insulin-resistant cells requiring elevated insulin levels for effective sugar metabolism. The gut microbiota and obesity share a close connection, and probiotic strains like *Lactobacillus pentosus* GSSK2 and *Lactobacillus plantarum* GS26A can contribute to obesity reduction [287]. Probiotics significantly enhance cytokine production and adiponectin levels compared with lyophilized single- or multi-strain formulations [288].

With an elevation in *Akkermansia* and *Prevotella* abundance, probiotics such as *Lactobacillus plantarum* L-14-derived exopolysaccharides activate the SIRT1-IRS1-Akt and GLUT2 pathways, improving lipid and glucose metabolism in insulin-resistant mice [289]. The TLR2-AMPK signaling pathway is activated by postbiotics, exemplified by exopolysaccharides from *Lactobacillus plantarum* L-14, inhibiting immature cell development into mature adipocytes and regulating body weight and lipid profiles in mice [290].The *Lactobacillus brevis* long-chain polyphosphate may alleviate intestinal inflammation and enhance intestinal barrier function by triggering the extracellular-regulated protein kinase (ERK) signaling pathway [291]. Muramyl dipeptide mitigates obesity-induced insulin resistance by targeting nucleotide-binding oligomerization domain-containing protein 2 (NOD2) and interferon regulatory factor 4 (IRF4) [239]. The overexpression of NLRC3 enhances colonic epithelial barrier integrity by increasing TNF receptor-associated factor 6 (TRAF6)-mediated ZO-1 and occludin expression. Butyrate improves the intestinal barrier in type 2 diabetic mice by upregulating GPR43 expression and stimulating NLCR3 in a TRAF6-dependent manner [292]. In NAFLD mice, butyrate restores intestinal barrier function by increasing ZO-1 expression and mitigates metabolic disorders and intestinal epithelial impairment in type 2 diabetic mice by promoting insulin secretion without compensatory hyperplasia in pancreatic β cells [293]. Postbiotics increaseantidiabetic activity by increasing insulin production, WAT Browning, glucose homeostasis, and insulin resistance (Figure 4).

### 8.5. Anti-Inflammatory

Inflammation serves as the initial response of an organism to infection, and several inflammatory disorders, including inflammatory bowel disease (IBD), arthritis, gastritis, asthma, atherosclerosis, and chronic inflammation, are associated with adverse side effects. Dysregulation of the nuclear factor kappa-light-chain-enhancer of activated B cells (NF-κB) and/or mitogen-activated kinase (MAPK) pathways, primarily involved in cell cycle regulation, is a common characteristic feature of these diseases [282,294,295]. A substantial body of evidence suggests the effectiveness of probiotics in alleviating the signs and symptoms of IBD and other inflammatory gastrointestinal diseases. The main molecular targets of the substances present in these probiotics are presumed to be the NF-κB and/or MAPK pathways. Bioactive compounds derived from probiotics emerge as a promising therapeutic strategy for mitigating excessive and/or prolonged inflammatory reactions [296,297,298].

MAPK signaling pathways, including c-Jun N-terminal kinases (JNKs), extracellular signal-regulated kinases (ERKs), and p38 MAP kinases, significantly influence cellular responses such as apoptosis, cytokine production, the regulation of proliferation, and inflammatory responses [299]. Postbiotics induce a robust response in inflammatory pathways by modulating the transcription of pro-inflammatory genes and the expression levels of pro-inflammatory proteins (Figure 4). However, the impact of postbiotics on the inflammatory response is contingent on factors like origin, bacterial species, strains, cell-free supernatant (CFS), and heat-killed bacteria (HKB). Both anti-inflammatory and immunostimulatory effects have been demonstrated, suggesting a heterogeneous structure and composition of different postbiotic media [86,300,301,302].

Inhibiting NF-κB nuclear translocation, IκB phosphorylation, and proteasomal degradation are three mechanisms through which postbiotics from CFS and HKB exert anti-inflammatory effects [303,304,305]. CFS from *L. rhamnosus* CNCM I-4036 enhances the expression of the IκB component NFKBIA, reinforcing the sequestration of NF-κB in the cytoplasm. Given that a similar set of pattern recognition receptors (PRRs) activates both the NF-κB and MAPK pathways, downstream processes may be impacted simultaneously, attenuating the inflammatory response by reducing NF-κB activity and MAPK activation [9]. Similar results were observed with isolated postbiotic factors, including 8.7 kDa proteins from *L. plantarum* 10hk2 CFS, DNA obtained from *L. rhamnosus* LGG, HKB from *Weissella cibaria* JW15, and CFS from *L. salivarius* MG4265 [2,121,306].

The injection of *L. reuteri* extract into mice accelerated wound healing, dependent on the activation of the PI3K/AKT/-catenin/TGF-1 pathway. Additionally, the CSF of *L. fermentum*, by inhibiting the PI3K/Akt/mTOR pathway, reduces the induction of H_2_O_2_-induced senescence in mouse adipocytes [307,308]. In monocytic cells, lipoteichoic acid (LTA) from *L. plantarum* exhibits anti-inflammatory effects and reduces the generation of TNF-α [72,309]. A crucial factor influencing the production of anti-inflammatory actions is the modification of postbiotic structure. For instance, in *L. rhamnosus* GG, the removal of D-alanine and substitution with glucosyl substitutions increased anti-inflammatory activity in murine colitis models [310,311]. In human intestinal cells, the elongation factor Tu (eFTu) and chaperone protein GroeL from *L. johnsonii* La1 stimulate IL-8 secretion in a CD14-dependent manner. CD14, acting as a co-receptor, is believed to amplify signals by inducing TLR4 endocytosis and activating TRIF-dependent pathways, resulting in the production of type I IFNs [312,313]. Probiotic-derived oligonucleotides such as gDNA and CpG motifs dramatically reduce LPS-induced IL-6 mRNA levels in RAW264.7 macrophages. *L. plantarum* gDNA pretreatment decreases the pro-inflammatory effect of LPS in THP-1 monocytic cells [170,314,315].

## 9. Conclusions and Future Perspectives

Since 2013, international research projects on postbiotics have seen a considerable increase. This review discusses a number of postbiotic topics, including historical viewpoints, significant developmental milestones, and noteworthy characteristics.The production, purification, and characterization of postbiotics, as well as their classification and the role of specific compounds, such as proteins, fatty acids, peptides, bacteriocins, enzymes, organic acids, extracellular vesicles, exopolysaccharides, and antioxidants, in the food industry are discussed. Given their unique properties, postbiotics exhibit promise as tools to control pathogenic microorganisms and function as antioxidants, anticancer, anti-diabetic, and anti-inflammatory agents. The therapeutic effects and mechanisms of postbiotics as antiviral, antibacterial, antioxidants, anticancer, and anti-inflammatory agents are presented. Although postbiotics exhibit pronounced effects on the food and health industries, further research is essential to establish standardized ways to evaluate the quantitative effects in a more reasonable, scientific, and accurate manner. Studies should also delve into the effects of postbiotics on health and their specific processes, necessitating in-depth investigation into the biological response of metabolites and host–postbiotic interactions using different omics approaches. This will bolster the wholesome and long-term growth of postbiotic preparations. Despite the scarcity of studies on postbiotic applications in the food industry and medicine, larger, higher-quality, and more rigorous trial data are required to demonstrate their benefits. Several countries have incorporated postbiotics into their national legislation, making goods available to the public despite the absence of defined global regulatory standards. This study contributes valuable standards for the evaluation of postbiotics, including their definition, mechanism, efficacy, and safety.

While postbiotic therapy’s safety and potential hazards remain incompletely studied and understood, further multicenter research is essential to establish outcomes and safety profiles for different postbiotics. Numerous metabolic, clinical, mechanistic, and biomedical studies are needed to identify new types of postbiotics, determine safe doses, and elucidate compound chemical structures. Understanding the mechanisms of immunomodulation by food is crucial for advancing and creating novel health solutions in the future.

## Figures and Tables

**Table 1:** Source, components, types, and functional aspects of postbiotics.

Bacteria	Derived Postbiotics	Functional Effects	References
*Bifidobacteriumlongum*	Bacterial lysates	Reduce cholesterol	Shin et al. [170]
		Anti-inflammation and antibacterial activity	Martorell et al. [171]
	Lipoteichoic acid	Anti-obesity	Balaguer et al. [172]
	Exopolysaccharides	Antioxidant	Inturri et al. [173]
		Immunomodulation	Inturri et al. [174]
		Anti-inflammation	Schiavi et al. [175]
*Lactobacillus* sp.		Anti-inflammation	Sungur et al. [176]
*Lactobacillus paracasei*		Reduce cholesterol	Bhat and Bajaj [177]
*Lacticaseibacillus rhamnosus*	Peptidoglycan	Immunomodulation	Kolling et al. [178]
*Lactobacillus plantarum*	Lipoteichoic acid	Immunomodulation	Kim et al. [179]
*Lactobacillus acidophilu*, *Lactobacillus reuteri*, *Lactobacillus plantarum*			Matsuguchi et al. [180]
*Lactobacillus paracasei*		Anti-inflammation	Wang et al. [181]
*Lactobacillus paracasei*	Bacterial lysates	Anti-obesity and reduce cholesterol	Osman et al. [182]
*Lacticaseibacillus rhamnosus*		Preventing alcoholic liver disease	Wang et al. [183]
*Lactobacillus casei*		Immunomodulation and anti-inflammation	Compare et al. [184]
*Lacticaseibacillus rhamnosus*		Antibacterial activity	Gao et al. [185]
*Lactobacillus amylovorus*		Anti-obesity and reduce cholesterol	Nakamura et al. [186]
*Lactobacillus plantarum*	Extracellular vesicles	Anti-inflammation	Haoet al., 2021 [187]
*Lacticaseibacillus rhamnosus*, *Lactobacillus acidophilu*	Cell-free supernatants	Anti-inflammation	Maghsood et al., 2018 [188]
*Lactobacillus* sp., *Bifidobacterium*sp.	Cytoflora (Brand)	Immunomodulation	Barros et al., 2020 [189]
*Lactobacillus* sps., *Nyaditumresea*, *Mycobacterium* sp.	Lacteol (Brand)		
*Bacillus velezensis*	Bacterial lysates	Immunomodulation and antibacterial activity	Mi et al. [190]
*Bacillus subtilis*	Polysaccharides	Anti-diabetic activity	Ghoneim et al. [191]
*Bacillus licheniformis*			Dahech et al. [192]
*Bacillus licheniformis*	Extra cellular vesicles	Anticancer activity	Gurunathan et al., 2023 [193]
*Bacillus coagulans*	Metabolites	Antioxidant	Jensen et al., 2017 [194]
*Saccharomyces cerevisiae*		Health advantages	Chan and Liu, 2022 [195]
*Saccharomyces cerevisiae*		Anti-inflammation	Jensen et al., 2007 [196]
*Faecalibacterium prausnitzii*	Bacterial lysates	Anti-inflammation	Sokol et al., 2008 [33]
*Clostridium butyricum*	Bacterial lysates	Anticancer activity	Chen et al., 2020 [197]
*Bifidobacterium breve*, *Streptococcus thermophilus*	Metabolites	Anti-inflammation	Menard et al., 2005 [198]
*Peanibacillus mucilaginosus*	Exopolysaccharides	Antioxidant	Liang et al., 2016 [199]
*Butyricicoccus pullicaecorum*	Butyric acid	Anti-inflammation	Geirnaert et al., 2017 [200]
*Enterococcus faecium*	Exopolysaccharides	Reduce cholesterol	Bhat and Bajaj, 2018 [201]
*Akkermansia muciniphila*	Inactivated bacteria	Anti-obesity	Depommier et al., 2020 [202]
*Methylococcus capsulatus*	Bacterial lysates	Anti-diabetic and immunomodulation	Jensenet al., 2021 [203]
*Bacteroides thetaiotaomicron*	Outer membrane vesicles	Anti-inflammation	Durant et al., 2020 [204]
TMAO (Brand)	Metabolites	Anticancer activity	Wang et al., 2022 [205]
*Pseudomonas aeruginosa*	Outer membrane vesicles	Antibacterial and anti-biofilm activity	Gurunathan et al., 2023 [167]
